# Mitochondrial DNA and traumatic brain injury

**DOI:** 10.1002/ana.24116

**Published:** 2014-03-01

**Authors:** Harry Bulstrode, James A. R. Nicoll, Gavin Hudson, Patrick F. Chinnery, Valentina Di Pietro, Antonio Belli

**Affiliations:** ^1^ University College London Cancer InstituteLondon; ^2^ Clinical Neurosciences, Division of Clinical and Experimental SciencesUniversity of SouthamptonSouthampton; ^3^ Wellcome Centre for Mitochondrial ResearchInstitute of Genetic Medicine, Newcastle UniversityNewcastle upon Tyne; ^4^ Division of Neurotrauma and NeurodegenerationUniversity of BirminghamBirmingham; ^5^ National Institute for Health Research Surgical Reconstruction and Microbiology Research CentreUniversity Hospitals BirminghamBirminghamUnited Kingdom

## Abstract

**Objective:**

Traumatic brain injury (TBI) is a multifactorial pathology with great interindividual variability in response to injury and outcome. Mitochondria contain their own DNA (mtDNA) with genomic variants that have different physiological and pathological characteristics, including susceptibility to neurodegeneration. Given the central role of mitochondria in the pathophysiology of neurological injury, we hypothesized that its genomic variants may account for the variability in outcome following TBI.

**Methods:**

We undertook an analysis of mitochondrial haplogroups in a large, well‐characterized cohort of 1,094 TBI patients. A proportional odds model including age, brain computed tomography characteristics, injury severity, pupillary reactivity, mitochondrial haplogroups, and *APOE* was applied to Glasgow Outcome Score (GOS) data.

**Results:**

mtDNA had a significant association with 6‐month GOS (*p* = 0.008). Haplogroup K was significantly associated with favorable outcome (odds ratio = 1.64, 95% confidence interval = 1.08–2.51, *p* = 0.02). There was also a significant interaction between mitochondrial genome and age (*p* = 0.002), with a strong protective effect of both haplogroups T (*p* = 0.015) and K (*p* = 0.017) with advancing age. We also found a strong interaction between *APOE* and mitochondrial haplogroups (*p* = 0.001), indicating a protective effect of haplogroup K in carriers of the *APOE* ε4 allele.

**Interpretation:**

These findings reveal an interplay between mitochondrial DNA, pathophysiology of TBI, and aging. Haplogroups K and T, which share a common maternal ancestor, are shown as protective in TBI. The data also suggest that the *APOE* pathways interact with genetically regulated mitochondrial functions in the response to acute injury, as previously reported in Alzheimer disease. Ann Neurol 2014;75:186–195

Traumatic brain injury (TBI) is a leading cause of death and disability across all ages and all populations. Its incidence continues to rise owing to increasing road use in developing countries and aging in the Western World; by 2020 the World Health Organization predicts that TBI will represent the third leading cause of morbidity and mortality worldwide. Understanding the variability in response to injury between individuals promises to be key to advances in clinical management. Existing studies have demonstrated detrimental outcome associated with the apolipoprotein E ε4 allele,^1^ and there is evidence for allele‐specific differences in outcome for other genes, including catechol‐o‐methyltransferase (*COMT*), dopamine D2 receptor (*DRD2*), phosphoprotein p53 (*TP53*), and the calcium channel alpha‐1 subunit (*CACNA1*).^2^ The mitochondrial genome has received little attention, despite mitochondria playing a central role in the pathophysiology of TBI. However, mitochondrial DNA (mtDNA) haplogroups have been widely reported as factors for degenerative pathologies and neurodegenerative conditions, including Parkinson disease (PD), Alzheimer disease (AD), Huntington disease, and motor neuron disease.^3^ For example, in PD, haplogroups J, T, U, and K, which share a common maternal ancestor distinct from the more common haplogroup H, have been shown by independent studies, and subsequent meta‐analyses, to confer a significant reduction in the risk of developing the condition.^4^ Associations have also been identified between mtDNA haplogroups and longevity; for example, haplogroups K, J, U, and D are overrepresented in centenarians,^6^ and a recent animal study by Gilmer et al indicated an age‐related increase in mitochondrial dysfunction following injury.^10^

The mtDNA encodes factors that are key to the mechanisms of cell damage and survival after neurological injury. Therefore, we reasoned that, as reported for neurodegenerative disorders, mtDNA haplogroups may account for some of the individual variability in resilience to TBI.

## Patients and Methods

### Participants

The study cohort (1,094 patients) was recruited from consecutive TBI admissions to the regional Neurosurgical Unit for the West of Scotland at the Institute of Neurological Sciences, Glasgow between 1996 and 1999. Consent and source DNA (buccal swab or blood sample) were obtained according to local research ethics committee (REC) permissions as previously detailed.^11^ This study originally set out to analyze the effect of apolipoprotein E (*APOE*) polymorphism on TBI outcome; the original consent form allowed for the samples to be retained for future genetic analysis of novel factors. Subsequent reanalysis of the existing DNA samples for the purposes of this study was approved by the Southampton and South West Hampshire Research Ethics Committee (REC reference 09/H0502/124).

### Clinical Features

Patient demographics, mechanism of injury, clinical presentation, key clinical events (raised intracranial pressure, seizures, sepsis, systemic complications, and surgery), and radiological and operative findings were collected prospectively by the study investigators. The computed tomography (CT) appearances at presentation were classified according to the Marshall system.^12^

### Outcome

Patients were contacted by mail and telephone at 6 months after injury. Answers to a structured questionnaire were obtained by staff unaware of the patient's status in the acute stage and of the results of genotyping. Proxy interviews were utilized wherever the patient was unable to answer the questionnaire in person. The medical records were also reviewed to collect mortality data. The patient's ability to conduct activities at home and outside, including shopping, travelling, working, and leisure activities, and the extent of any family disruption was used to evaluate their Glasgow Outcome Scale (GOS).^13^

Some survivors were functioning at a severely disabled level even prior to injury; these were allocated to the favorable outcome group if they recovered to their previous state, or to the unfavorable group if they were left with increased disability and dependency. Patients for whom the available information was insufficient to discriminate between moderate disability and good recovery were allocated to the favorable group if it was clear that they were independent in society.

### mtDNA Haplogrouping

The mtDNA haplogroup was determined from buccal swabs or blood samples by analysis with polymerase chain reaction and restriction fragment length polymorphism according to methods described by Torroni et al.^14^ By this procedure, each mtDNA was ascribed to 1 of the 5 haplogroups (H, J, K, T, and U) specific to European populations. Uncommon haplogroups and mtDNAs that were not classifiable within a haplogroup were grouped as “others.”

### Exclusions and Missing Data

From the initial study cohort, 91 were determined to have a chronic subdural hematoma as opposed to an acute head injury and were therefore excluded, leaving 1,003 eligible patients. Twenty‐two of these were missing follow‐up data; additionally, in 101 patients haplogrouping was not achieved. The 880 remaining patients are analyzed here.

### Statistical Analysis

Statistical analysis was undertaken using SPSS software version 20 (IBM Solutions, Armonk, NY).

An ordinal regression analysis (proportional odds model) was carried out to analyze the effect of mitochondrial haplogroups on outcome, in accordance with established statistical principles in this field.^15^ The predictors were selected based on the variables of the “core + CT” model of the International Mission on Prognosis and Analysis of Clinical trials in Traumatic brain injury (IMPACT; http://www.tbi‐impact.org).^16^ These variables include age, motor score of the Glasgow Coma Scale, pupillary reactivity, hypoxia, hypotension, Marshall CT classification grade, presence of traumatic subarachnoid hemorrhage, and presence of epidural hematoma. This model and its predictive variables have received external validation in large cohort studies.^17^ The ordinal regression analysis included the polychotomous term “mtDNA haplogroup” (H, J, T, U, K, and others), the dichotomous term “*APOE* genotype” (ε4/non‐ε4), and all the above terms, except “epidural hematoma,” which was not available for our cohort. *APOE* genotype was available for every case from the original study.^11^

For the purpose of the analysis, the 5‐point GOS was collapsed into a 4‐point scale, pooling together death and vegetative outcomes. The collapsed GOS scale is commonly used in TBI studies, as a vegetative outcome is not generally regarded an improvement from death. The remaining 3 points on this scale were severe disability, moderate disability, and good outcome.

We also analyzed the interaction between mtDNA haplogroups and age, and between mtDNA haplogroup and *APOE* by fitting the interaction terms “age by mtDNA haplogroup” and “*APOE* genotype by mtDNA haplogroup” into the model.

The ordinal regression analysis assumptions and goodness of fit were tested and found to be valid. A *p* value of <0.05 was considered significant.

## Results

### mtDNA and Response to Injury

The haplogroup distribution was in line with comparable UK studies,^4^ with the predominant European haplotype H accounting for 40.5% of this study population. Mitochondrial haplogroup distribution, along with key demographic and clinical parameters, is shown in Table 1.

**Table 1:** Patient Demographics and Admission Characteristics by Mitochondria Haplogroup Frequency Followed by Percentage of Total for Given Haplogroup Except Where Stated

Characteristic	Haplogroup						
	H	J	T	U	K	Other	All
Frequency (% of total)	357 (40.5)	122 (13.8)	78 (8.9)	146 (16.6)	74 (8.4)	103 (11.8)	880 (100)
Sex							
M	297	96	62	115	65	83	718
F	60	26	16	31	9	20	162
Age, mean yr [SD]	35 [20.7]	38 [24.5]	34 [21.5]	34 [22.5]	34 [20.6]	33 [21.6]	35 [21.7]
Motor score of GCS (%)							
No response	30 (8.4)	11 (9)	6 (7.7)	18 (12.3)	4 (5.4)	4 (3.9)	73 (8.3)
Extending to pain	9 (2.5)	2 (1.6)	2 (2.6)	8 (5.5)	4 (5.4)	5 (4.9)	30 (3.4)
Abnormal flexion	10 (2.8)	3 (2.5)	3 (3.8)	1 (0.7)	1 (1.4)	5 (4.9)	23 (2.6)
Withdrawing to pain	33 (9.2)	9 (7.4)	6 (7.7)	12 (8.2)	2 (2.7)	5 (4.9)	67 (7.6)
Localizing to pain	74 (20.7)	23 (18.9)	20 (25.6)	26 (17.8)	18 (24.3)	14 (13.6)	175 (19.9)
Obeying commands	196 (54.9)	70 (57.4)	40 (51.3)	77 (52.7)	43 (58.1)	67 (65)	493 (56)
Not assessable	5 (1.4)	4 (3.3)	1 (1.3)	4 (2.7)	2 (2.7)	3 (2.9)	19 (2.2)
Pupil reactivity (%)							
Both	310 (88)	105 (86)	70 (90)	117 (81)	65 (88)	86 (84)	753 (86)
One	18 (5)	4 (3)	3 (4)	13 (9)	5 (7)	7 (7)	50 (6)
Neither	26 (7)	13 (11)	5 (6)	15 (10)	4 (5)	9 (9)	72 (8)
CT grade (%)							
N/A	2 (1)	1 (1)	0	1 (1)	1 (1)	0	5 (1)
1	94 (26)	39 (32)	19 (24)	34 (15)	13 (6)	22 (21)	221 (25)
2	132 (37)	44 (36)	31 (40)	58 (40)	38 (51)	38 (37)	341 (39)
3	22 (6)	7 (6)	7 (9)	8 (6)	5 (7)	4 (4)	53 (6)
4	3 (1)	1 (1)	0	0	1 (1)	0	5 (1)
5	84 (24)	22 (18)	17 (22)	37 (25)	11 (15)	36 (17)	207 (24)
6	20 (6)	8 (7)	4 (5)	8 (6)	5 (7)	3 (3)	48 (6)
Traumatic SAH (%)							
No	283 (79.3)	99 (81.1)	61 (78.2)	108 (74)	57 (77)	83 (80.6)	691 (78.5)
Yes	74 (20.7)	23 (18.9)	17 (21.8)	38 (26)	17 (23)	20 (19.4)	189 (21.5)
Hypoxia (%)	7 (2)	3 (2.5)	2 (2.6)	1 (3)	0 (0)	5 (2.1)	18 (2)
Hypotension (%)	19 (5.3)	7 (5.7)	7 (9)	10 (6.8)	6 (8.1)	5 (4.9)	54 (6.1)
ApoE (%)							
ε2 or ε3	241 (67.5)	85 (69.7)	50 (64.1)	99 (67.8)	50 (67.6)	63 (61.2)	588 (66.8)
ε4	116 (32.5)	37 (30.3)	28 (35.9)	47 (32.2)	24 (32.4)	40 (38.8)	292 (33.2)

CT = computed tomography; F = female; GCS = Glasgow Coma Scale; M = male; N/A = not available; SAH = subarachnoid hemorrhage; SD = standard deviation.

The predictive value (*R*^2^) of the IMPACT model is 0.35, with the remainder of the variability thought to be accounted for either genetically or by other biological factors yet to be identified.^19^ After adding mitochondrial haplogroups to the predictive terms of the IMPACT model, we found a significantly predictive effect of mtDNA genotype on the 6‐month GOS (*p* = 0.008).

When haplogroups were considered individually, patients who possessed the K variant had significantly better outcome than those who did not (odds ratio [OR] = 1.64, 95% confidence interval [CI] = 1.08–2.51, *p* = 0.02). However, individually, there was no significant association between 6‐month outcome and haplogroups H, J, T, and U, and all other uncommon variants. The outcome data are shown in Table 2. As expected, all other predictive terms were found to be significant (Table 3), with the exception of *APOE* and hypoxia (only 18 patients—2% of the sample—were recorded to have suffered a hypoxic episode).

**Table 2:** Outcomes 6 Months after Head Injury According to Mitochondrial Haplogroup

Parameter	GOS	Haplogroup						
		H	J	T	U	K	Other	All
Frequency (% of total)		357 (40.5)	122 (13.8)	78 (8.9)	146 (16.6)	74 (8.4)	103 (11.8)	880 (100)
Outcome (%)								
Dead	1	45 (13)	18 (15)	7 (9)	15 (10)	6 (8)	10 (10)	101 (12)
Vegetative	2	13 (4)	4 (3)	2 (3)	7 (5)	2 (3)	5 (5)	33 (4)
Severe disability	3	64 (18)	13 (11)	12 (15)	31 (21)	11 (15)	23 (22)	154 (18)
Moderate disability	4	92 (26)	41 (34)	24 (31)	41 (28)	18 (24)	21 (20)	237 (27)
Good recovery	5	143 (40)	46 (38)	33 (42)	52 (36)	37 (50)	44 (43)	355 (40)

GOS = Glasgow Outcome Scale.

**Table 3:** Model Effects

Parameter	Category	Chi‐Square	*df*	*F*	OR (95% CI)	*p*
mtDNA haplogroup, overall effect		15.601	5	3.120		0.008
	H				1.57 (0.84–2.96)	
	J				1.2 (0.55–2.63)	
	T				1.23 (0.52–2.88)	
	U				1.14 (0.55–2.39)	
	K				0.21 (0.07–0.56)	
	Other				0.83 (0.38–1.82)	
Age		132.52	1	132.52	1.04 per year (1.03–1.04)	0.000
Motor score of GCS		46.739	6^a^	7.790		0.000
	No response				1 REFERENCE	
	Extending to pain				1.35 (0.64–2.87)	
	Abnormal flexion				1.6 (0.73–3.55)	
	Withdrawing to pain				0.58 (0.34–0.93)	
	Localizing to pain				0.57 (0.34–0.93)	
	Obeying commands				0.3 (0.18–0.48)	
	Not assessable				0.23 (0.09–0.61)	
CT Marshall grade		59.238	5	11.856		0.000
	6				1 REFERENCE	
	5				0.38 (0.22–0.67)	
	4				0.24 (0.03–1.8)	
	3				0.39 (0.19–0.78)	
	2				0.22 (0.13–0.38)	
	1				0.52 (0.27–0.84)	
Traumatic SAH		10.149	1	10.149		0.001
	Absent				1 REFERENCE	
	Present				1.57 (1.17–2.11)	
Hypotension [blood pressure < 90mmHg within the first 24 hours]		4.002	1	4.4002		0.046
	Absent				1 REFERENCE	
	Present				1.8 (1.2–2.7)	
Hypoxia [PO_2_ < 8kPa or SaO_2_ < 85% in the first 24 hours]		0.945	1	0.945		0.331
	No hypoxia				1 REFERENCE	
	Confirmed hypoxia				0.67 (0.3–1.5)	
Pupillary reactivity		9.112	2	4.556		0.011
	Both reacting				1 REFERENCE	
	Only 1 reacting				1.71 (1.05–2.78)	
	Neither reacting				1.83 (1.09–3.08)	
ApoE genotype [ε4 vs ε2 or ε3]		0.507	1	0.507		0.476
	ε4				1 REFERENCE	
	ε2/ε3				0.82 (0.41–1.63)	
Interaction between mtDNA haplogroup and age		13.439	5	2.688		0.02
Interaction between mtDNA haplogroup and *APOE* ε4		21.35	5	4.27		0.001

Effects of predictors of the ordered regression analysis on the collapsed Glasgow Outcome Scale. Chi‐quare, degrees of freedom (*df*), *F* statistics, odds ratio (OR), and significance are shown. OR < 1 favors better outcome. As mtDNA haplogroup is a polynomial term, the ORs for this parameter indicate the odds of better outcome for carriers of each haplogroup compared to noncarriers of the same haplogroup.

a Includes the 6 possible motor scores and the category “not assessable.”

CI = confidence interval; CT = computed tomography; GCS = Glasgow Coma Scale; SAH = subarachnoid hemorrhage.

### Interaction between mtDNA and Aging

**Figure 1:**
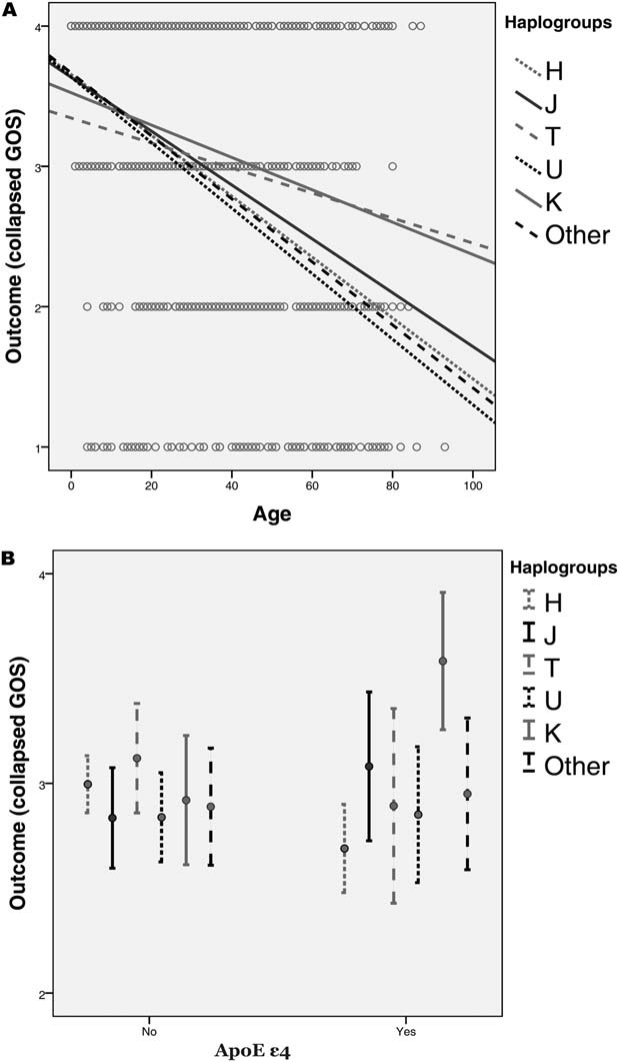
Effect of mtDNA haplogroups on outcome in relation to age (A) and *APOE* genotype (B). Panel A shows the linear regression lines of each haplogroup on a scatter plot of outcome versus age. Each circle represents a case. Haplogroups K and T clearly have different slopes from the other haplogroups, indicating that age, which is a strong determinant of outcome in traumatic brain injury, has a comparatively smaller effect in patients who possess these haplogroups. Panel B illustrates the interaction between mtDNA haplogroups and *APOE* genotype on outcome, illustrated by the 95% confidence interval of the collapsed Glasgow Outcome Scale (GOS) score (1 = dead or vegetative, 2 = severe disability, 3 = moderate disability, 4 = good outcome) by mtDNA haplogroups and *APOE* ε4 possession.

Mitochondrial dysfunction is a shared feature of TBI and aging; therefore, we reasoned that mtDNA haplogroups might differentially modulate the effect of aging as a determinant of TBI outcome. The inclusion of the interaction term “age by haplogroup” in the model revealed a strong interaction between age and mtDNA haplogroups (*p* = 0.002). Possessing either haplogroup K or T significantly mitigated the negative effect of aging on outcome (*p* = 0.017 and *p* = 0.015, respectively), as shown by Figure 1A.

These findings are consistent with the evidence from epidemiological studies showing over‐representation of haplogroup K in longevity and under‐representation of haplogroups K and T in neurodegenerative conditions such as PD.^4^

### Interaction between mtDNA and APOE Genotype

*APOE* has several known relevant roles after brain injury, from the maintenance of vascular integrity and function of the blood–brain barrier to protection against oxidative stress. *APOE* has 3 isoforms that have allele‐specific effects (E2 > E3 > E4) in protecting neuronal cell lines from oxidative cell death.^22^ An interaction between *APOE* polymorphisms and mtDNA haplogroups in TBI has been described for AD in a cohort of 213 patients.^23^ In view of this, we analyzed a possible interaction between these factors in our cohort. We found this interaction to be highly significant (*p* = 0.001). Specifically, patients who carried the *APOE* ε4 allele had significantly better outcome if they also possessed the mtDNA K haplogroup (OR = 5.86, 95% CI = 2.14–17.44, *p* = 0.002), as shown by Figure 1B.

## Discussion

The response to TBI is multifactorial; clinical presentation, physiological condition, and clinical management are determinants of outcome, but genetic factors are also hypothesized to play a key role. Few genomic associations have been identified in the field of TBI. The *APOE* gene is arguably the most extensively studied in this area, and recently cytokine polymorphisms have also been found to influence the outcome of TBI in the same cohort of patients studied here.^24^

**Figure 2:**
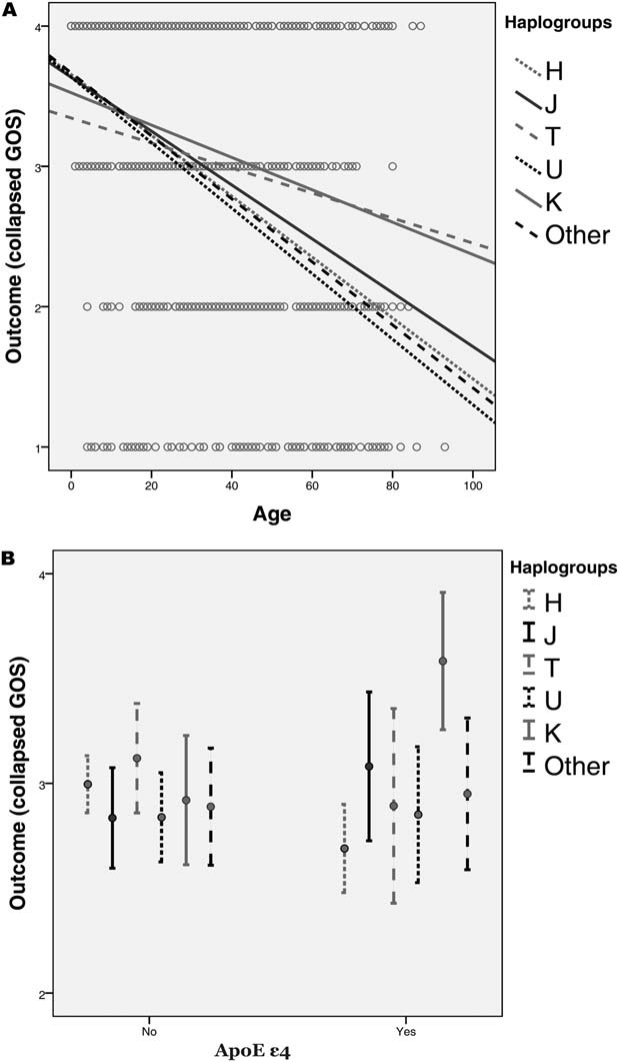
The human mitochondrial DNA and known and putative genetic location of mutations associated with neurological conditions. ATP = adenosine triphosphate; MELAS = mitochondrial encephalomyopathy, lactic acidosis, and strokelike episodes; MERRF = myoclonic epilepsy with ragged red fibers; MND = motor neuron disease; NADH = nicotinamide adenine dinucleotide; TBI = traumatic brain injury. Adapted from the Free Software Foundation and licensed under the GNU Free Documentation License.

Although most mitochondrial proteins are encoded by nuclear DNA, mtDNA encodes key components; these include 13 key subunits of the electron transport chain, as well as essential components of the translational machinery (Fig 2). MtDNA haplogroup is a determinant of the efficiency of oxidative phosphorylation (OXPHOS), with substantial physiological and pathophysiological implications. In crude terms, it is thought that haplogroups conferring more or less efficient OXPHOS may have evolved in response to selective pressures of food scarcity and cold climate, respectively.^25^ The differential susceptibility to age‐related neurodegenerative conditions demonstrated by different mtDNA haplogroups can then be framed in terms of the OXPHOS efficiency, and resulting rate of generation of reactive oxygen species (ROS), associated with individual haplogroups^4^ (see Fig 2). A similar argument has been proposed to explain the over‐representation of haplogroups K, J, U, and D in centenarians.^6^

TBI, across the entire spectrum of severity, imposes a metabolic stress associated with reduced OXPHOS capacity of neural tissue, and generation of ROS.^27^ Our results point to a protective effect of haplogroup K. The enzymatic components of the mitochondrial electron transport chain (ETC) encoded by haplogroup K are reported to be less tightly coupled than corresponding variants in other haplogroups, which reduces ROS production as a byproduct of adenosine triphosphate (ATP) synthesis.^20^ ROS are key mediators of secondary cell damage after a neurological insult, including TBI^29,30^; it is therefore unsurprising that a less tightly coupled ETC may confer a cell survival advantage after injury. Recent literature also suggests that downregulation of ATP synthesis after TBI may be a neuroprotective mechanism.^31^ This may also provide an explanation for a less tightly coupled ETC conferring a neuroprotective effect in the case of haplogroup K.

### Genomic Variants of mtDNA Differentially Mitigate the Effect of Aging on TBI Outcome

Aging causes a progressive loss of mitochondrial function and a reduction in OXPHOS enzyme activities in human tissues, including the brain.^33^ In addition, mitochondria are also both the source and the target of ROS damage, as mtDNA lacks protective histones and DNA repair enzymes.^34^ With aging, cells accumulate a progressively larger burden of mtDNA damage, which is preferentially, clonally amplified within cells. The mitochondrial energetic output therefore declines and ROS production increases, as does the propensity for apoptosis, leading to progressive cell loss and tissue function decline.

It is important to remember that age is a very strong determinant of outcome after TBI, with most studies showing a linear relationship between age and TBI mortality and disability after the 3rd decade of life.^19^ We therefore reasoned that mtDNA haplogroups might differentially affect resilience to TBI with advancing age. We found a strong mitigating effect of haplogroups K and T on the effect of aging on TBI outcome. The observed effect of haplogroup K is compatible with its reduced ROS output compared to other haplogroups.^20^ The effect of haplogroup T is less clear, but it is of note that, similarly to haplogroup K, haplogroup T has been found to be under‐represented in neurodegenerative conditions.^4^ It is somewhat surprising that we did not see a similar interaction between age and mtDNA haplogroup J, given the close genetic relationship between haplogroups J and T. Of note, these haplogroups are distinguished by different variants of nicotinamide adenine dinucleotide (NADH) dehydrogenase, another key enzyme of the respiratory chain.

### Haplogroup K Interacts with *APOE* ε4

We did not find an association between *APOE* genotype and outcome in our cohort. This is consistent with the findings of the original study from which our cohort was derived,^11^ despite some methodological differences between the 2 studies. However, we found a very strong interaction between *APOE* ε4 and mtDNA haplogroup K. A similar interaction has been reported by Carrieri et al for AD; this study found significant under‐representation of haplogroups K and U in ε4 carriers and concluded that these haplogroups might neutralize the harmful effect of the *APOE* ε4 allele.^23^ As illustrated by Figure 1B, in our cohort there is a strong effect of haplogroup K on outcome in patients who are *APOE* ε4 positive. For those who lack *APOE* ε4, haplogroup K appears to have no impact on outcome. The low ROS production associated with haplogroup K might be expected to mitigate the low efficiency of the antioxidant activity of *APOE ε4*,^35^ and this would seem to be an attractive possible mechanism of interaction for further study.

### The Role of Mitochondrial Genome in the Pathophysiology of TBI

Few studies to date have examined the role of the mitochondrial genome in TBI pathophysiology. In a small study, long‐term survivors of TBI were shown to have a lower prevalence of key mtDNA deletions than age‐matched controls, raising the possibility that free radical–induced accumulation of mtDNA damage may selectively influence the survival of mitochondria or their host.^36^ In another recent study of 336 patients by Conley et al, mitochondrial single nucleotide polymorphisms (SNPs) were found to be significantly associated with patient outcomes 1 year after injury^37^; only severe TBI patients managed with external ventricular drainage were eligible for this study, but the findings are nonetheless broadly compatible with ours. For example, Conley et al found the A10398G mitochondrial SNP, located within the NADH–ubiquinone oxidoreductase subunit‐3 (ND3) locus encoding part of complex I, to be associated with TBI outcome; the A10398 and 10398G alleles of this SNP were associated, respectively, with slower and faster recovery. The A10398 allele is found in haplogroup H and appears to increase the susceptibility to neurodegenerative and mental health disorders,^5^ whereas the 10398G allele is associated with haplogroups K and J, and is reported to exert a protective effect from the risk of PD. It is important to bear in mind that the modifying role of an mtDNA haplogroup on injury response or disease risk is most probably due to the synergistic action of a set of different polymorphisms rather than to the effect of a single polymorphism. Haplogroups J and K belong to widely diverging mitochondrial clades,^38^ which may explain the different results obtained for the 2 haplogroups in our cohort.

No effective neuroprotective drugs for TBI have emerged to date. The limited ability to explain the variability in response to injury has resulted in challenges in dealing with signal‐to‐noise ratio and consequent underpowering of many previous studies. Our findings have the potential to contribute to effective patient stratification in clinical trials, and mtDNA genomics should be included in future outcome prediction models, for example those developed on the basis of forthcoming comparative effectiveness research studies. Moreover, the molecular mechanisms of cell damage following TBI are still poorly understood; our findings shed further light on the pivotal role of mitochondria in the pathophysiology of TBI and advance our understanding of the molecular mechanisms regulated by mitochondrial genome.

The interpretation must accept certain limitations of the study. First, our cohort was drawn from neurosurgical admission, and the findings may not be generalizable to unselected admissions. However, the study included a large number of patients who were not severely injured, and so the bias is likely to be limited. Second, although the follow‐up rate was extremely high, data are incomplete in some participants, and mitochondrial genotyping was not successful on all samples, although there is no suggestion that test failures did not occur randomly. Third, outcome was assessed by GOS, which is a global measure of outcome; the possible effects of mtDNA haplogroups on the individual components of outcome (eg, neuropsychological health status, behavior, social interaction, and functional performance) would have added further dimensionality to our findings, although a much larger study would have been required to explore multiple parameters. Nevertheless, GOS remains the most widely used method of assessment of outcome in TBI and has several advantages. These include its applicability across all ranges of age, severity, and outcome; the high reliability and validity of assessment achieved by the structured approach used in this study; and its high degree of correlation with the results of specific assessments focused on limited aspects of the state of survivors (eg, cognitive, behavioral, and emotional sequelae) and of detailed multidimensional assessments of health and psychosocial state, such as the SF‐36 questionnaire.^39^ Finally, mitochondrial haplogroup frequencies vary greatly between populations, and the nuclear genetic background of subjects with haplogroups T and K may differ from the nuclear genetic background of other mtDNA haplogroups. Therefore, it is possible that further associations of mtDNA haplogroups with particular nuclear genotypes are responsible for our findings, rather than the mtDNA haplogroups themselves. This issue is likely to be minimized by the analysis being restricted to a cohort from a regional unit in Scotland.

Although our cohort had a representative distribution of mtDNA haplogroups with respect to Northern European populations, like all genetic studies, it would be advisable to validate our findings in different geographical regions and populations.

## Authorship

J.A.R.N. was involved in the design of the Glasgow *APOE* study that provided the prospective data set and patient population for this study. H.B., P.F.C., G.H., and A.B. designed the current study. H.B., V.D.P., and A.B. analyzed the data. H.B. and A.B. wrote the article. All authors contributed to the methodology of the study and interpretation of the findings, and reviewed the article.

## Potential Conflicts of Interest

Nothing to report.

## References

[1] Alexander S, Kerr ME, Kim Y, et al. Apolipoprotein E4 allele presence and functional outcome after severe traumatic brain injury. J Neurotrauma 2007;24:790–797 17518534 10.1089/neu.2006.0133

[2] Jordan BD. Genetic influences on outcome following traumatic brain injury. Neurochem Res 2007;32:905–915 17342413 10.1007/s11064-006-9251-3

[3] Lehman EJ, Hein MJ, Baron SL, Gersic CM. Neurodegenerative causes of death among retired National Football League players. Neurology 2012;79:1970–1974 22955124 10.1212/WNL.0b013e31826daf50 PMC4098841

[4] Pyle A, Foltynie T, Tiangyou W, et al. Mitochondrial DNA haplogroup cluster UKJT reduces the risk of PD. Ann Neurol 2005;57:564–567 15786469 10.1002/ana.20417

[5] van der Walt JM, Nicodemus KK, Martin ER, et al. Mitochondrial polymorphisms significantly reduce the risk of Parkinson disease. Am J Hum Genet 2003;72:804–811 12618962 10.1086/373937 PMC1180345

[6] Ross OA, McCormack R, Curran MD, et al. Mitochondrial DNA polymorphism: its role in longevity of the Irish population. Exp Gerontol 2001;36:1161–1178 11404057 10.1016/s0531-5565(01)00094-8

[7] Rose G, Passarino G, Carrieri G, et al. Paradoxes in longevity: sequence analysis of mtDNA haplogroup J in centenarians. Eur J Hum Genet 2001;9:701–707 11571560 10.1038/sj.ejhg.5200703

[8] Tanaka M, Gong JS, Zhang J, et al. Mitochondrial genotype associated with longevity. Lancet 1998;351:185–186 9449878 10.1016/S0140-6736(05)78211-8

[9] Niemi AK, Hervonen A, Hurme M, et al. Mitochondrial DNA polymorphisms associated with longevity in a Finnish population. Hum Genet 2003;112:29–33 12483296 10.1007/s00439-002-0843-y

[10] Gilmer LK, Ansari MA, Roberts KN, Scheff SW. Age‐related mitochondrial changes after traumatic brain injury. J Neurotrauma 2010;27:939–950 20175672 10.1089/neu.2009.1181 PMC2943941

[11] Teasdale GM, Murray GD, Nicoll JA. The association between APOE epsilon4, age and outcome after head injury: a prospective cohort study. Brain 2005;128(pt 11):2556–2561 16033781 10.1093/brain/awh595

[12] Maas AI, Hukkelhoven CW, Marshall LF, Steyerberg EW. Prediction of outcome in traumatic brain injury with computed tomographic characteristics: a comparison between the computed tomographic classification and combinations of computed tomographic predictors. Neurosurgery 2005;57:1173–1182; discussion 1182 16331165 10.1227/01.neu.0000186013.63046.6b

[13] Wilson JT, Pettigrew LE, Teasdale GM. Structured interviews for the Glasgow Outcome Scale and the extended Glasgow Outcome Scale: guidelines for their use. J Neurotrauma 1998;15:573–585 9726257 10.1089/neu.1998.15.573

[14] Torroni A, Huoponen K, Francalacci P, et al. Classification of European mtDNAs from an analysis of three European populations. Genetics 1996;144:1835–1850 8978068 10.1093/genetics/144.4.1835 PMC1207732

[15] McHugh GS, Butcher I, Steyerberg EW, et al. Statistical approaches to the univariate prognostic analysis of the IMPACT database on traumatic brain injury. J Neurotrauma 2007;24:251–258 17375989 10.1089/neu.2006.0026

[16] Steyerberg EW, Mushkudiani N, Perel P, et al. Predicting outcome after traumatic brain injury: development and international validation of prognostic scores based on admission characteristics. PLoS Med 2008;5:e165; discussion e165 18684008 10.1371/journal.pmed.0050165 PMC2494563

[17] Perel P, Arango M, Clayton T, et al. Predicting outcome after traumatic brain injury: practical prognostic models based on large cohort of international patients. BMJ 2008;336:425–429 18270239 10.1136/bmj.39461.643438.25 PMC2249681

[18] Lingsma H, Andriessen TM, Haitsema I, et al. Prognosis in moderate and severe traumatic brain injury: external validation of the IMPACT models and the role of extracranial injuries. J Trauma Acute Care Surg 2013;74:639–646 23354263 10.1097/TA.0b013e31827d602e

[19] Lingsma HF, Roozenbeek B, Steyerberg EW, et al. Early prognosis in traumatic brain injury: from prophecies to predictions. Lancet Neurol 2010;9:543–554 20398861 10.1016/S1474-4422(10)70065-X

[20] Wallace DC. A mitochondrial bioenergetic etiology of disease. J Clin Invest 2013;123:1405–1412 23543062 10.1172/JCI61398 PMC3614529

[21] Khusnutdinova E, Gilyazova I, Ruiz‐Pesini E, et al. A mitochondrial etiology of neurodegenerative diseases: evidence from Parkinson's disease. Ann N Y Acad Sci 2008;1147:1–20 19076426 10.1196/annals.1427.001

[22] Miyata M, Smith JD. Apolipoprotein E allele‐specific antioxidant activity and effects on cytotoxicity by oxidative insults and beta‐amyloid peptides. Nat Genet 1996;14:55–61 8782820 10.1038/ng0996-55

[23] Carrieri G, Bonafe M, De Luca M, et al. Mitochondrial DNA haplogroups and APOE4 allele are non‐independent variables in sporadic Alzheimer's disease. Hum Genet 2001;108:194–198 11354629 10.1007/s004390100463

[24] Waters RJ, Murray GD, Teasdale GM, et al. Cytokine gene polymorphisms and outcome after traumatic brain injury. J Neurotrauma 2013;30:1710–1716 23768161 10.1089/neu.2012.2792 PMC3796334

[25] Tranah GJ, Manini TM, Lohman KK, et al. Mitochondrial DNA variation in human metabolic rate and energy expenditure. Mitochondrion 2011;11:855–861 21586348 10.1016/j.mito.2011.04.005 PMC3998521

[26] Gomez‐Duran A, Pacheu‐Grau D, Lopez‐Gallardo E, et al. Unmasking the causes of multifactorial disorders: OXPHOS differences between mitochondrial haplogroups. Hum Mol Genet 2010;19:3343–3353 20566709 10.1093/hmg/ddq246

[27] Vagnozzi R, Marmarou A, Tavazzi B, et al. Changes of cerebral energy metabolism and lipid peroxidation in rats leading to mitochondrial dysfunction after diffuse brain injury. J Neurotrauma 1999;16:903–913 10547099 10.1089/neu.1999.16.903

[28] Vagnozzi R, Tavazzi B, Signoretti S, et al. Temporal window of metabolic brain vulnerability to concussions: mitochondrial‐related impairment—part I. Neurosurgery 2007;61:379–388; discussion 388–389 17762751 10.1227/01.NEU.0000280002.41696.D8

[29] Lifshitz J, Sullivan PG, Hovda DA, et al. Mitochondrial damage and dysfunction in traumatic brain injury. Mitochondrion 2004;4:705–713 16120426 10.1016/j.mito.2004.07.021

[30] Tavazzi B, Vagnozzi R, Signoretti S, et al. Temporal window of metabolic brain vulnerability to concussions: oxidative and nitrosative stresses—part II. Neurosurgery 2007;61:390–395; discussion 395–396 17806141 10.1227/01.neu.0000255525.34956.3f

[31] Di Pietro V, Amorini AM, Tavazzi B, et al. Potentially neuroprotective gene modulation in an in vitro model of mild traumatic brain injury. Mol Cell Biochem 2013;375:185–198 23242602 10.1007/s11010-012-1541-2

[32] Di Pietro V, Amin D, Pernagallo S, et al. Transcriptomics of traumatic brain injury: gene expression and molecular pathways of different grades of insult in a rat organotypic hippocampal culture model. J Neurotrauma 2010;27:349–359 19903084 10.1089/neu.2009.1095

[33] Bowling AC, Mutisya EM, Walker LC, et al. Age‐dependent impairment of mitochondrial function in primate brain. J Neurochem 1993;60:1964–1967 8473911 10.1111/j.1471-4159.1993.tb13430.x

[34] Wallace DC. A mitochondrial paradigm of metabolic and degenerative diseases, aging, and cancer: a dawn for evolutionary medicine. Annu Rev Genet 2005;39:359–407 16285865 10.1146/annurev.genet.39.110304.095751 PMC2821041

[35] Jofre‐Monseny L, Minihane AM, Rimbach G. Impact of apoE genotype on oxidative stress, inflammation and disease risk. Mol Nutr Food Res 2008;52:131–145 18203129 10.1002/mnfr.200700322

[36] McDonald RP, Horsburgh KJ, Graham DI, Nicoll JA. Mitochondrial DNA deletions in acute brain injury. Neuroreport 1999;10:1875–1878 10501524 10.1097/00001756-199906230-00014

[37] Conley YP, Okonkwo D, Deslouches S, et al. Mitochondrial polymorphisms impact outcomes after severe traumatic brain injury. J Neurotrauma 2014;31:34–41 23883111 10.1089/neu.2013.2855 PMC3880110

[38] Ghezzi D, Marelli C, Achilli A, et al. Mitochondrial DNA haplogroup K is associated with a lower risk of Parkinson's disease in Italians. Eur J Hum Genet 2005;13:748–752 15827561 10.1038/sj.ejhg.5201425

[39] Pettigrew LE, Wilson JT, Teasdale GM. Reliability of ratings on the Glasgow Outcome Scales from in‐person and telephone structured interviews. J Head Trauma Rehabil 2003;18:252–258 12802167 10.1097/00001199-200305000-00003

